# Genome Sequence of a Novel Archaeal Rudivirus Recovered from a Mexican Hot Spring

**DOI:** 10.1128/genomeA.00040-12

**Published:** 2013-01-15

**Authors:** Luis E. Servín-Garcidueñas, Xu Peng, Roger A. Garrett, Esperanza Martínez-Romero

**Affiliations:** a Department of Ecological Genomics, Center for Genomic Sciences, National University of Mexico, Cuernavaca, Morelos, Mexico; b Department of Biology, Archaea Centre, University of Copenhagen,Copenhagen, Denmark

## Abstract

We report the consensus genome sequence of a novel GC-rich rudivirus, designated SMR1 (*Sulfolobales* Mexican rudivirus 1), assembled from a high-throughput sequenced environmental sample from a hot spring in Los Azufres National Park in western Mexico.

## GENOME ANNOUNCEMENT

Thermophilic archaeal viruses have been isolated from thermal terrestrial sites, revealing an incredibly large viral diversity (1–3). Mexico contains active volcanoes and geothermal areas extending along the Trans-Mexican Volcanic Belt (TMVB) (4). However, the thermophilic viral diversity present within the TMVB hot springs remains unexplored. Here, we present the consensus genome sequence of a novel rudivirus recovered by iterative *de novo* read mapping and assembly from the metagenome of a hot spring located along the northern edge of the TMVB.

Aqueous sediment samples were collected from an acidic hot spring (pH 3.6 and 65°C) located at Los Azufres, Mexico, in March 2009. The DNA was purified using the UltraClean microbial DNA and UltraClean Mega soil DNA kits (MoBio Laboratories, Inc., Carlsbad and Solana Beach, CA). The metagenomic DNA was sequenced with an Illumina GAIIx platform producing 36-bp paired-end reads with 300-bp inserts representing 216 Mbp. The reads were assembled *de novo* using Velvet 1.2.07 (5). The contigs with overrepresented coverage were verified by BLASTX searches to be of viral origins. The reads were mapped to the viral contigs using Maq 0.7.1 (6), and the mapping reads were reassembled to eliminate gaps. The coding sequences were predicted using GeneMark.hmm 2.0 (7) and were manually verified using Artemis (8).

The sequence coverage of the 27,431-bp double-stranded DNA genome was 240-fold. The presence of an inverted terminal repeat (1,240 bp), characteristic of the linear rudiviral genomic termini, indicated that the genome was complete or almost complete. The G+C content of 46.6% was higher than the 25 to 39% content of the four rudiviral genomes characterized previously (9–11). The host is likely to be a member of the order *Sulfolobales*, the sequences of which dominated the metagenome. Moreover, the G+C content of *Sulfolobales* Mexican rudivirus 1 (SMR1) was similar to those of the *Metallosphaera* genomes (∼45%). Thirty-seven open reading frames (ORFs) were identified, 19 of which have putative homologs in the other characterized rudiviruses; this strongly supports SMR1 being a member of the *Rudiviridae* family. Common annotated gene products include the major coat protein, three minor structural proteins, two glycosyl transferases, a clustered regularly interspaced short palindromic repeats (CRISPR)-associated Cas4-like protein, a putative replication protein, a Holliday junction helicase, a Holliday junction resolvase, an *S*-adenosylmethionine-dependent methyltransferase, and a putative transcriptional regulator. The seven other shared rudiviral proteins were not assigned functions.

Three additional ORFs show sequence similarities to archaeal ORFs, including one carrying a zinc finger SWIM domain and a predicted CopG domain. Four other ORFs contain domains related to the thioredoxin-like superfamily and the GTP-binding proteins, as well as a ribbon-helix-helix protein and a nop25 domain-containing protein. Eight additional ORFs showed no significant matches. Interestingly, three ORFs were related to viral ORFs of the *Lipothrixviridae* family. Of the 6,000,792 environmental reads, 183,365 (3.05%) mapped to the consensus viral genome and 115 candidate single nucleotide polymorphisms (SNPs) were detected by Maq.

In conclusion, despite the large geographical distance from the locations of other sequenced rudiviruses, SMR1 retained a core set of conserved rudiviral genes that were inferred to be important for the viral life cycle.

### Nucleotide sequence accession number. 

The genome sequence was deposited in GenBank under the accession no. JX944686.
